# The complete mitochondrial genome of the Basidiomycete edible fungus *Hypsizygus marmoreus*

**DOI:** 10.1080/23802359.2018.1532343

**Published:** 2018-10-30

**Authors:** Ying-Ying Wu, Jun-Jun Shang, Yan Li, Chen-Li Zhou, Di Hou, Jia-Li Li, Qi Tan, Da-Peng Bao, Rui-Heng Yang

**Affiliations:** National Engineering Research Center of Edible Fungi, Ministry of Science and Technology (MOST), Key Laboratory of Edible Fungi Resources and Utilization (South), Ministry of Agriculture, Institute of Edible Fungi, Shanghai Academy of Agricultural Sciences, Shanghai, China

**Keywords:** *Hypsizygus marmoreus*, Agaricales, Lyophyllaceae, mitochondrial genome

## Abstract

The complete mitochondrial genome of the edible fungus *Hypsizygus marmoreus* was published in this paper. It was determined using Pacbio and Illumina sequencing. The complete mitochondrial DNA (mtDNA) is 106,417 bp in length with a GC content of 31.74%, which was the fourth large mitogenome in Agaricales. The circular mitogenome encoded 67 protein-coding genes and one ribosomal RNAs (rns). Among these genes, 13 conserved protein-coding genes were determined in the genome, including 6 subunits of NAD dehydrogenase (nad1-4, 4L and 6), three cytochrome oxidases (cox1-3), one apocytochrome b (cob) and three ATP synthases (atp6, apt 8 and apt 9). The phylogenic analysis confirmed that *H. marmoreus* (Lyophyllaceae) clustered together with *Tricholoma matsutake* (Tricholomataceae).

## Introduction

*Hypsizygus marmoreus* (Peck) H. E. Bigelow, belonging to Agaricales, is a commercial edible mushroom. Because of its high nutritional and medicinal value, it is very popular in East Asian regions, including China and Japan (Wu et al. 2015). Some studies have shown that mushrooms are promising foods in improving human health and preventing diseases. In previous investigations, steroids, sphingolipids, proteins and polyisoprenepolyols were isolated from the fruiting bodies of this fungus (Akihisa et al. 2005; Jung et al. 2008; Krasnopolskaya et al. 2008; Lee et al. 2012). Some studies indicated that strains of *H. marmoreus* show abundant diversities in morphological and genetic characters. PCR-based molecular markers and inter-simple sequence repeat (ISSR) have been widely used in genetic studies of *H. marmoreus* (Wang et al. 2009; Lee et al. 2012; Qiu et al. 2013). However, there were no enough polymorphic sites for identifying different strains. The mitogenome reported here might provide important genetic information for further studies on phylogeny, strain conservation, identification or discrimination of the strains.

**Figure 1.**
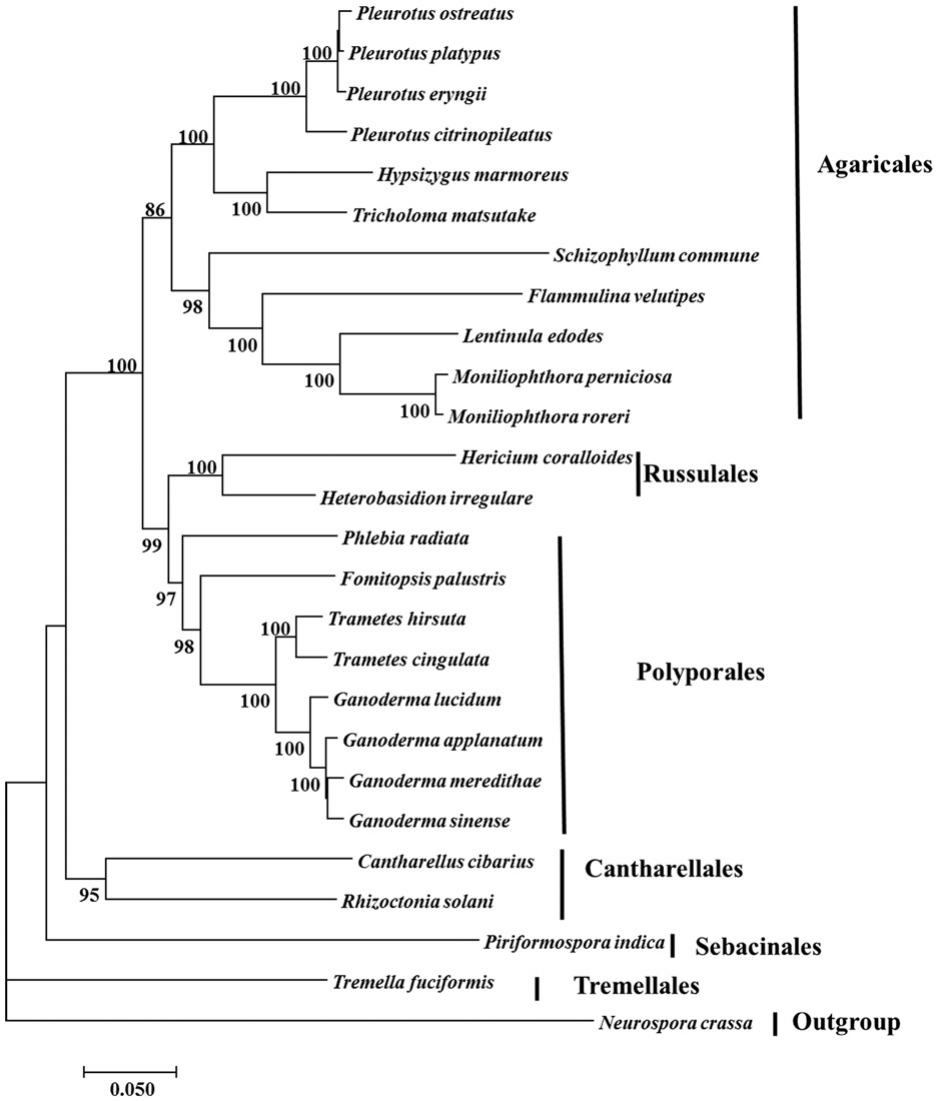
Neighbour-joining tree of 26 species of Agaricomycotina conducted using MEGA 7.0 (Kumar et al. 2016) based on concatenated amino acid sequences of 13 mitochondrial protein-coding genes, including atp6, atp8, atp9, cob, cox1, cox2, cox3, nad1, nad2, nad3, and4, nad4L, nad5 and nad6. All the sequences were aligned using Clustal X (Thompson et al. 2010). The 25 other species used in this study were listed following: *Cantharellus cibarius* (NC_020368), *Flammulina velutipes* (NC_021373), *Fomitopsis palustris* (NC_034349), Hericium coralloides (NC_033903), *Ganoderma applanatum* (NC_027188), *Ganoderma lucidum* (NC_021750), *Ganoderma meredithae* (NC_026782), *Ganoderma sinense* (NC_022933), *Heterobasidion irregulare* (NC_024555), *Lentinula edodes* (NC_018365), *Moniliophthora perniciosa* (NC_005927), *Moniliophthora roreri* (NC_015400), *Pleurotus citrinopileatus* (NC_036998),*Pleurotus ostreatus* (NC_009905), Pleurotus platypus (NC_036999), *Phlebia radiata* (NC_020148), *Rhizoctonia solani* (HF546977), *Schizophyllum commune* (NC_003049), *Serendipita indica* (FQ859090), Trametes hirsuta (NC_037239), Tremella fuciformis(NC_036422), *Trichosporon asahii* var. asahii (MT: JH925097), *Trametes cingulata* (NC_013933) and *Tricholoma matsutake* (NC_028135). *Neurospora crassa* (NC_026614) was served as an outgroup. The percentages of replicate trees in which the associated taxa clustered together in the bootstrap test (1000 replicates) were shown next to the branches.

Monokaryoitc strain (F4 isolated from dikaryotic strain B5 deposited at Institute of Edible Fungi, Shanghai Academy of Agricultural Sciences) isolation, genomic DNA extraction, strategies of sequencing (Illumina and Pacbio), sequencing processes, genome assembly and annotation, phylogenetic analysis were conducted according to the methods published previously (Yang et al. 2016a, 2016b, 2017; Wan et al. 2018). Assembly of a total of 258,199 Pacbio subreads (2,860,968,946 bp) and 67,421,744 high quality Illumina reads (9,955,446,419 bp) resulted in 106,417 bp of mitogenome which might be the fourth large mitogenome in Agaricales (*Agaricus bisporus* – 133 kb; *Lentinula edodes* – 116 kb; *Moniliophthora perniciosa* – 109 kb) (Férandon et al. 2013; Yang et al. 2017). The GC content was 31.74%. The circular mitogenome encoded 69 putative protein-coding genes and one ribosomal RNAs (rns). Thirteen conserved protein-coding genes encoded 6 subunits of NAD dehydrogenase (nad1-4, nad4L and nad 6), three cytochrome oxidases (cox1-3), one apocytochrome b (cob) and three ATP synthases (atp6, apt 8 and apt 9). Nine introns invaded into three genes, i.e. cob (1 intron), cox1 (7 introns) and cox2 (1 intron). These introns mainly belong to group IB. The 22 tRNA genes covered 19 standard amino acids, with the following three having two tRNAs each: two trnL (trnL-uaa and trnL-uag), two trnR (trnR-ucg and trnR-ucu) and two trnS (trnS-gcu and trnS-uga) while the remaining amino acids were each represented by only one tRNA gene. This genome could not encode Alanine. As shown in Figure 1, the phylogenetic analysis confirmed that *H. marmoreus* (Lyophyllaceae) was a member of Agaricales and clustered together with *Tricholoma matsutake* (Tricholomataceae). It was in agreement with previous study, Tricholomatoid clade included four families, the Tricholomataceae s. str., Lyophyllaceae, Entolomataceae and Mycenaceae, and the Catathelasma clade (Matheny et al. 2006; Zhao et al. 2017). The evolutionarily relationship among Agaricales, Russulales, Polyporales, Cantharellales and Sebacinales was in agreement with results of previously study (Hibbett 2006; Garcia-sandoval et al. 2011; Zhao et al. 2017). The mitogenome of *H. marmoreus* would provide new insights into understanding the phylogeny and evolution of Lyophyllaceae and Agaricales.

## Data Availability

This genome sequence has been deposited at NCBI (http://www.ncbi.nlm.nih.gov/) under the accession no. MH746465.
